# A curriculum-integrated sun safety intervention to improve adolescent skin health knowledge in an independent girls’ secondary school

**DOI:** 10.1371/journal.pone.0350659

**Published:** 2026-06-26

**Authors:** Rachael S. Jefferson, Kelly M. Bentley-Spuur, Kenneth Chinkwo, Clare L. Singh, Simon Board, Philip L. Tong, Minh T. Chau

**Affiliations:** 1 School of Education, Charles Sturt University, Thurgoona, New South Wales, Australia; 2 School of Dentistry and Medical Sciences, Charles Sturt University, Wagga Wagga, New South Wales, Australia; 3 Kambala, Rose Bay, New South Wales, Australia; 4 Department of Dermatology, St Vincent’s Hospital, Darlinghurst, New South Wales, Australia; Mae Fah Luang University School of Anti Aging and Regenerative Medicine, THAILAND

## Abstract

Australia has one of the world’s highest melanoma incidences among youth. Adolescents often under-prioritise sun protection due to sociocultural factors, for example, the perceived attractiveness of tanned skin and a sense of invulnerability. This study reports on a curriculum-integrated sun safety intervention implemented in a single independent girls’ secondary school, a context that provides important insights but also limits generalisability to mixed-gender and other school settings. High school students (aged 13–15) participated in sun safety workshops integrated into their Personal Development, Health, and Physical Education (PDHPE) curriculum. Sun protection knowledge, attitudes toward tanning, and self-reported protective behaviours were assessed via surveys administered pre-intervention, immediately post-intervention, and five months later. Students’ sun safety knowledge increased significantly post-intervention (p < 0.001), and remained higher than baseline knowledge at 6 months, indicating substantial knowledge retention. Participants reported more frequent sunscreen use and protective behaviours after the program. The intervention also reduced the appeal of tanning, fewer students agreed that “a suntan looks good” after participating. Notably, all students regardless of ethnicity (Caucasian, Asian, Other), showed knowledge gains, but variations were observed. Students from historically underserved ethnic backgrounds started with lower baseline knowledge and exhibited slightly smaller improvements aligning with prior findings that sun awareness has differed across racial/ethnic group. These findings suggest that an engaging, curriculum-integrated sun safety program can produce immediate and sustained improvements in adolescents’ sun protection knowledge and attitudes within a girls’ secondary school context. While the single-site design limits broader generalisation, the results highlight the potential value of embedding sun safety education within school curricula. Future research should examine the effectiveness of this intervention across multiple school sites, including co-educational and diverse educational settings, to strengthen evidence for scalability and policy implementation aimed at long-term skin cancer prevention.

## Introduction

Australia has the highest melanoma (skin cancer) rates in the world [1]. Melanoma is the third most commonly diagnosed cancer in Australia and the most common cancer diagnosed in individuals aged 20–39 years of age [1]. In 2024, it is predicted that 16,800 Australians will be diagnosed with melanoma, and there will be 1,300 melanoma deaths. Importantly, overexposure to ultraviolet (UV) radiation from the sun and sunburn (a visible sign of UV radiation damage to the skin) is responsible for 95% of all melanoma lesions and is largely preventable [2].

Sun safety messages of the past, such as the “Slip-Slop-Slap” campaign of the 1980s, increased awareness of melanoma prevention [1], however, this messaging has not overtly changed the desirability of having a tan or the culture of tanning in Australia. A subsequent 2007–2011 research-informed campaign by the Cancer Institute NSW, the ‘Dark Side of Tanning’, reported that concerns about the risk of skin cancer in 13–24-year-olds were outweighed by the desirability of a “healthy” tan [3]. The Cancer Council in 2019 advises that the habits of teenagers 12–17 in protecting themselves against UV radiation and sunburn have not changed since 2007, and there has been no significant decrease in rates of sunburn [2]. This may be due to sociocultural norms that promote an idea of ideal skin tone [4]. This is concerning as epidemiological studies support an age relationship and increased risk of melanoma with history of sunburn during childhood [1,5].

Girls have been identified as at greater risk where peer norms and gender-targeted messaging related to tanning as an acceptable and popular behaviour have been found to indirectly affect sun safety [6,7]. Adolescents are known to place great importance on social acceptance and cosmetic appearance [8], and in this context, the sun-related activities of young women have been reported to be engaged towards meeting physical and psychosocial needs [8,9]. These sociocultural norms and the belief that having a tan is healthy, the perceived attractiveness of having a tan, body image, peer norms, and fashion are all known barriers to protective sun-safe behaviours [6,10–13]. Longstanding barriers such as these, combined with the contemporary impact of social media [7] and social influencers in glamorising intentional tanning, and misinformation concerning the use of sunscreens, have significantly impacted sun-safe messaging.

Being at the highest risk for melanoma and with high-risk behaviours, poor behavioural change levels and unavoidable social media influence, teenagers are an ongoing priority for sun-safe messaging to decrease intentional and unintentional tanning. In their study, Geller et al. (2003) demonstrated the potential for sun protection education that was brief and standardised to be effectively presented in school curricula. The study reported increased knowledge and positive sun protection intentions [14]. Other research supports the potential for the success of high school educational programs on skin cancer prevention, which for greatest impact should be based on evidence-based learning and teaching strategies [15]. It has also been reported that presentations delivered as multiple units rather than short-duration presentations were more effective [16].

There is indeed a longstanding belief that skin cancer incidence may be reduced by addressing sun safety determinants in the school years [6]. A 2023 systematic review of evidence-based high school melanoma prevention curricula reported a significant increase in knowledge, behavioural change, and attitude changes [15]. Other studies have documented limited changes in sun safety behaviours compared to improved sun safety knowledge [16] and acknowledged that sun safety protection was inconsistently translated into sun-safe behaviours [17].

Behavioural change rates were higher where adolescents received multiple sun protection information sources, including school messages, compared to fewer sources [18]. Interestingly, in another study, traditional printed health promotion media was reported to be more impactful among young adults in increasing knowledge compared to online platforms, reflecting the need for a mixed approach to sun safety education and messaging [19]. Stage-matched interventions have also been identified as beneficial in the context of sun safety promotion [20].

However, the development and delivery of skin cancer prevention messaging has greatest impact on younger adolescents when engaged by their near or older peers in contrast to non-peers (including teachers, parents, and other adults) [21–23]. Peer leaders can be used to ensure that prevention messaging is relevant, reflecting the needs of their peers [21]. Engaging slightly older, “aspirational” peers who are perceived as credible, further uses the power of social networks to shape behaviour. This approach is further grounded in the Theory of Active Involvement, which evidences a self-reflective process for those involved in development and delivery with subsequent changes in their own attitudes and intentional behaviours [23].

This research aims to understand the effectiveness of dedicated skincare/sun safety workshops and syllabus implementation in improving Australian high school students’ understanding and of skin care and protection within an independent girls’ school context. It presents a targeted opportunity to engage students on an authentic and individual level (including peer leadership), supported by experiential learning instead of theoretical learning alone. Given that the literature reports adolescents’ ongoing poor sun-safe behaviour, exploring the effectiveness of differing approaches to messaging is warranted.

The secondary aims of this study are to report students’ knowledge and understanding of skin care and protection before engaging in dedicated sun safety workshops; the immediate impact of the workshop on students’ knowledge and understanding of skincare and protection after engaging in the workshops and to investigate the retention of knowledge and understanding of skin care and protection.

While adolescents broadly remain a priority group for sun safety education, this study is situated within a single independent girls’ secondary school. Girls have been identified as a particularly high-risk group due to sociocultural norms, peer influences, and gendered messaging around tanning and appearance. As such, this research provides a context-specific examination of sun safety education within a girls’ school setting, rather than aiming for immediate generalisation across all school types.

## Materials and methods

### Workshop and survey design

The dedicated sun safety workshops took place in an independent girls’ day and boarding school in Rose Bay, Sydney, Australia, which caters to students from Preparatory (around 4 years old) through to Year 12. The selection of a single independent girls’ school enabled an in-depth, contextually responsive intervention design but necessarily limits the generalisability of findings to other school types, including co-educational, government, or boys’ school settings. Workshops were to be classroom-based, informed by the New South Wales (NSW), Personal Development, Health and Physical Education (PDHPE) syllabus, and initiated and led by the classroom teacher. Participants, Year 8 students were initially introduced to the proposed workshop in this study through a year group presentation describing the ‘Skin Safety & Sun Care Workshop in Schools’ at the school site. Students were subsequently sent a bulk year group email, which offered the voluntary opportunity to form a working group to help co-design the workshop to be held at the school on 29 August 2024. A working group of 15 students was formed, and they provided feedback on all aspects of the workshop planning including: making the survey questions student friendly; providing feedback on the integration for the Anne Gately “SunBurnt” book and what content engages most with them; interrogating the workshop stations to make sure they were going to achieve the best outcome for the students, and discussing the impact of influencers.

### Workshops

The workshop had theoretical and practical components. The theoretical component included five stations (8 minutes each) and a one-hour group performance presentation by a guest speaker with lived experience of skin cancer who conducted a group presentation based around the key topics: Skin Structure and Genetics, Myths, Types of Skin Cancer and Historical and Cultural Perspectives. Each of these topics was included after considering the current PDHPE syllabus resources (Sun and UV at School) [24], and importantly through advice from the student co-design team.

The practical component consisted of five stations. Station 1 had a UV camera for students to explore their own skin; Station 2 explored sun screen contents of a variety of sunscreens (and formulations) and provided sampling opportunities; Station 3 gave students the opportunity to undertake an Escape room challenge aimed at developing strategies for evaluating digital sources including mobile apps and they were then given five minutes to solve whether a TikTok was valid using the SIFT method (Stop, Investigate the source, Find better coverage, and Trace claims: a four-step strategy for quickly assessing online sources) [25]; Station 4 reported the medical consequences of the three major types skin cancer using one survivor and a plastic surgeon to explain a variety of experiences; Station 5 involved skin self-care where students were able to try on a range of products designed to repair skin including after sunburn. Students were given sample bags at the conclusion of the workshop, provided by L’Oréal.

### Questionnaire

To measure the outcome of the workshop, a survey method was chosen as the most cost-effective method of collecting data. Using a Likert scale, a *Pre Workshop Survey* asked students 22 questions and a *Post Workshop Survey* (used immediately after the workshops and again 5 months after this to verify retention levels) asked students 21 questions (see S1 Appendix). All 16 matched questions employed a five-point Likert scale. Responses were converted to numerical values ranging from 1 (‘Strongly Disagree’) to 5 (‘Strongly Agree’). To ensure consistent comparison across all three time points (Time 1: Pre Workshop Survey; Time 2: Post Workshop Survey; Time 3: Post Workshop Survey – 5-month delay), the final statistical analysis was conducted on a matched dataset of 16 consistent survey items. These 16 matched survey items were intentionally developed to align with the core outcomes of the NSW PDHPE syllabus (see S1 Appendix) and were organised into thematic content areas corresponding to the design of the in-school workshop stations (Table 1). This approach ensured that the evaluation directly measured knowledge gains relevant to curriculum outcomes, specifically relating to health literacy, protective behaviours, and the influence of contextual factors on health.

**Table 1:** Alignment of matched survey items with thematic domains and NSW PDHPE syllabus-aligned workshop topics.

Survey Question Number	Survey Theme (MANOVA Grouping)	Corresponding Workshop Topic (Syllabus-Aligned)
Q1	Clinical Awareness	Skin Structure and Genetics
Q2	Clinical Awareness	Skin Structure and Genetics & UV Camera
Q4	Clinical Awareness	Emerging Technology and Early Diagnosis
Q14	Clinical Awareness	Emerging Technology and Early Diagnosis
Q6	Product Knowledge	Sunscreen Contents
Q7	Product Knowledge	Sunscreen Contents
Q8	Product Knowledge	Sunscreen Contents
Q3	Sun Safety Knowledge	UV Camera and Sunscreen Contents
Q9	Sun Safety Knowledge	Health Literacy (UV Index)
Q15	Sun Safety Knowledge	Skin Care Station
Q10	Self-check Skills	Health Literacy (Online Content Accuracy)
Q13	Self-check Skills	Medical Consequences (ABCDE Self-Check)
Q11	Cultural Awareness	Historical and Cultural Influences
Q12	Cultural Awareness	Historical and Cultural Influences
Q5	Anatomy & Damage	Skin Structure and Genetics
Q16	Skin Care Habits	Skin Care Station

The questionnaire was purpose-designed for this study and was not adapted from an existing validated instrument. Development was informed by the study aims and relevant literature on students’ sun safety behaviours and attitudes. Draft items were reviewed and piloted by all the researchers who have combined expertise in health, physical education, cancer research, dermatology, and diagnostic radiography mixed methods methodology to establish content validity and ensure clarity and age-appropriateness. Minor revisions were made following this review. Internal consistency reliability was examined for multi-item scales using Cronbach’s alpha. Constructs measured using single items were not subject to reliability analysis.

Due to small subgroup sizes, ethnicity categories were collapsed for analysis, with “Other” and “Prefer not to say” responses combined into a single category to ensure sufficient cell sizes for MANOVA.

In terms of directionality, the questionnaire items were structured such that a higher numerical score indicated a higher level of knowledge, confidence, or agreement with the desired sun-safe principle. These scores were subsequently treated as interval-level variables for statistical analysis. As survey completion was voluntary, the statistical analysis used a repeated measures design. Each student was assigned a unique identifier to enable accurate matching of responses across the three survey time points . Therefore, the analysis was restricted to a final matched dataset of 31 students who completed all three surveys (pre-workshop, post-workshop, and five-month follow-up). Any entries from students who did not complete all three time points were excluded from the final analysis to ensure the integrity of the repeated measures design.

Written consent was obtained from parents, and students were able to participate in three surveys via a link provided by the Charles Sturt University Spatial Data Analysis Network (SPAN). The pre workshop survey was made available to students on the 26th of August 2024; the post-workshop survey at the end of the workshop from the 29th of August until the 19th of September 2024 and a second delayed post-workshop survey from the 19th of February until the 12th of March 2025. The two post workshop surveys were available for a period of three weeks; with a reminder to participate forwarded at the end of the second week. Completion of the surveys was voluntary.

### Ethics

This study, “Can dedicated workshops and associated syllabus implementation be used to improve high school students’ knowledge and understanding of skin care and protection?” was approved by Charles Sturt University’s Human Research Ethics Committee, Protocol number H24204. Ethical approval was granted until 08/02/2027. All procedures were conducted in accordance with the university’s guidelines for research involving human participants. Written informed consent was obtained from all participating students and their parent/guardian as required.

### Statistical analysis

All analyses were conducted using IBM SPSS Statistics for Windows, Version 30.0.0.0 (IBM Corp., Armonk, NY). Survey responses were measured using Likert scales and converted to numerical values ranging from 0 (Strongly Disagree) to 5 (Strongly Agree). These were treated as interval-level variables. Descriptive statistics were computed for all items.

Normality was assessed using Shapiro-Wilk tests and Q-Q plot inspection. Levene’s test was used to assess homogeneity of variance. Only items meeting assumptions were included in parametric testing. A repeated measures design was used, with Time (pre-workshop, post-workshop, and five-month follow-up) as the within-subjects factor, and Ethnicity (Caucasian, Asian, Other) as the between-subjects factor. A multivariate analysis of variance (MANOVA) using Wilks’ Lambda was conducted to examine main and interaction effects across all 16 items. To facilitate the interpretation of the statistical findings, the 16 survey items were grouped into thematic categories. This thematic categorisation helps simplify the interpretation of the results and allows for a clearer understanding of which aspects of sun safety and skin cancer knowledge are most affected by the intervention. For survey questions showing significant effects, Bonferroni-adjusted pairwise comparisons were used to identify where differences occurred across timepoints and ethnic groups.

## Results

### Participant characteristics

Participant responses to the 16 survey questions used for workshop evaluation analysis, were selected based on consistency of completion across all three survey timepoints: pre-workshop, immediate post-workshop, and five-month post-workshop follow-up. The pre-workshop survey was completed by 81 students, the post-workshop by 80 students, and the five-month follow-up by 57 students. Each student was assigned a unique identifier to enable matching across surveys. This process resulted in a final matched dataset of 31 students who completed all three timepoints. Table 2 presents the descriptive statistics (mean and standard deviation) for each survey item across the three timepoints.

**Table 2:** Survey themes and descriptive statistics by timepoint (pre-, post-, and 5-month follow-up).

Q#	Survey Question	Theme	Pre-workshop (mean±SD)*	Post-workshop (mean±SD)*	Five-month post-workshop (mean±SD)*
1	I understand that skin cancer risk can be passed from family members genetically.	Clinical awareness	3.42 ± 0.67	3.74 ± 0.51	3.61 ± 0.56
2	I am aware of the potential dangers of UV damage on the skin.	Clinical awareness	3.60 ± 0.56	3.74 ± 0.51	3.71 ± 0.53
3	I know how to apply sunscreen to prevent sunburn.	Sun safety knowledge	3.40 ± 0.67	3.71 ± 0.59	3.61 ± 0.56
4	I can explain how early diagnosis and the use of technology (skin damage recognition devices such as UV cameras, high-resolution cameras and total body photography) can demonstrate the damaging impact of skin cancer.	Clinical awareness	2.58 ± 0.85	3.65 ± 0.61	3.45 ± 0.57
5	I can confidently provide information about the structure of my skin.	Anatomy & damage	2.68 ± 0.87	3.67 ± 0.54	3.39 ± 0.61
6	I understand the different types of sunscreen available and how they differ in effectiveness (physical, chemical, broad spectrum: absorb or reflect UVB and UVA rays).	Product knowledge	2.45 ± 1.18	3.71 ± 0.53	3.42 ± 0.76
7	I know what Sun Protection Factor (SPF) stands for.	Product knowledge	3.40 ± 0.62	3.65 ± 0.55	3.55 ± 0.62
8	I understand what SPF differences mean (e.g., 30 versus 50 SPF).	Product knowledge	3.43 ± 0.68	3.76 ± 0.51	3.51 ± 0.56
9	I understand the UV index and use it to help protect myself from the sun.	Sun safety knowledge	3.19 ± 0.60	3.68 ± 0.60	3.52 ± 0.57
10	I know how to determine the accuracy of online content regarding sun safety, including on apps and social media.	Self-check skills	2.81 ± 1.01	3.71 ± 0.53	3.35 ± 0.55
11	I am aware of ethnicity differences in terms of what a healthy skin colour looks like.	Cultural awareness	3.23 ± 0.67	3.68 ± 0.54	3.29 ± 0.74
12	I know that skin types differ in the protection they offer.	Cultural awareness	3.17 ± 0.75	3.61 ± 0.56	3.29 ± 0.74
13	I can confidently explain the methods for performing a self-skin examination using the A (Asymmetry) B (Border) C (Colour) D (Diameter) E (Evolution) method.	Self-check skills	1.48 ± 1.15	3.67 ± 0.54	3.3 ± 0.70
14	I can explain how early diagnosis can limit the damaging impact of skin cancer.	Clinical awareness	3.03 ± 1.02	3.22 ± 0.99	3.03 ± 0.95
15	I know what to do after I get a sunburn to help heal quickly.	Sun safety knowledge	3.23 ± 0.72	3.67 ± 0.61	3.03 ± 0.95
16	I know how to implement a daily skincare routine to care for my skin and protect it from sun damage.	Skin care habits	2.55 ± 0.85	3.68 ± 0.60	3.29 ± 0.69

*Items scored 0–5 (0 = Strongly disagree, 5 = Strongly agree); values are mean±SD; higher scores indicate greater knowledge/awareness/behaviour as stated.

Students self-identified their ethnicity using predefined categories. For statistical analysis, responses were collapsed into three analytic groups: Caucasian, Asian, and Other. The “Other” category included students who selected “Other (please specify)” as well as those who selected “Prefer not to say,” in order to retain these participants in the repeated-measures analysis while protecting anonymity and maintaining adequate group sizes. Of these 31 students, 13 identified as Caucasian, 9 as Asian, and 7 as Other. An additional 2 students selected “Prefer not to say” and 6 entries initially listed as “Other (please specify)” were included under the “Other” category for analysis. This approach is consistent with prior educational research where sample sizes limit subgroup analysis. Individual socioeconomic status data were not collected as part of this study. As a result, economic status could not be included in participant-level analyses, which represents a limitation when considering broader generalisability.

### Workshop evaluation results

The aggregated numerical dataset used to generate all statistical results in this study is available in S1 File. The results presented below reflect outcomes observed within this specific school context and should be interpreted accordingly. A repeated measures MANOVA using Wilks’ Lambda was conducted to assess changes in student responses across three time points (pre-workshop, post-workshop, and five-month follow-up) on 16 survey items. The within-subjects factor was Time (three time points), and the between-subjects factor was Ethnicity (Caucasian, Asian, and Other). Table 3 provides a summary of the MANOVA results.

**Table 3:** Multivariate analysis of variance (MANOVA) results for time, ethnicity, and interaction effects.

Survey Item	Time Effect	Ethnicity Effect	Pre vs Post	Post vs 5-Month
Q1: Genetic risk		F(2,77)=7.41, p = .001		
Q2: UV damage awareness		F(2,77)=5.95, p = .004		
Q3: Sunscreen application		F(2,77)=3.43, p = .037		
Q4: UV camera technology	F(2,77)=15.62, p < .001		Mean Diff = −1.00, p < .001	
Q5: Sunscreen types	F(2,77)=13.27, p < .001		Mean Diff = −0.96, p < .001	Mean Diff = −0.69, p < .001
Q6: UV impact on skin	F(2,77)=16.32, p < .001		Mean Diff = −1.29, p < .001	Mean Diff = −1.00, p < .001
Q7: Skin tone perceptions		F(2,77)=9.66, p < .001		
Q8: SPF meaning		F(2,77)=3.40, p = .038		
Q9: SPF differences	F(2,77)=3.60, p = .032			
Q10: UV index	F(2,77)=8.41, p < .001		Mean Diff = −0.82, p < .001	Mean Diff = −0.51, p = .032
Q11: Online content evaluation	F(2,77)=3.90, p = .024		Mean Diff = −0.43, p = .046	
Q12: Ethnicity & skin tone	F(2,77)=2.90, p = .061	F(2,77)=4.46, p = .015		
Q13: ABCDE method	F(2,77)=44.40, p < .001		Mean Diff = −2.21, p < .001	Mean Diff = −1.84, p < .001
Q14: Early diagnosis		F(2,77)=3.11, p = .050		
Q15: Sunburn aftercare	F(2,77)=4.26, p = .018			
Q16: Daily skincare routines	F(2,77)=15.87, p < .001	F(2,77)=4.63, p = .013	Mean Diff = −1.18, p < .001	Mean Diff = −0.76, p < .001

There was a significant main effect of time on the combined dependent variables, Wilks’ Λ = .196, F(32, 124) = 4.867, p < .001, indicating that student responses changed significantly across time points. A significant main effect of ethnicity was also observed, Wilks’ Λ = .389, F(32, 124) = 2.335, p < .001, suggesting that responses varied by ethnic group. However, the time by ethnicity interaction was not significant, Wilks’ Λ = .437, F(64, 244.995) = 0.901, p = .684, indicating that the pattern of change over time was consistent across the three ethnic groups.

### Between-subject effects

Univariate tests revealed several significant effects of time and ethnicity on individual post-questionnaire items. A main effect of time was observed across multiple survey items, indicating significant differences in student responses between the pre-workshop, post-workshop, and five-month follow-up. Students showed increased understanding over time in the use of technology to detect skin damage (Q4: F(2,77) = 15.62, p < .001), sunscreen types (Q5: F(2,77) = 13.27, p < .001), skin structure and UV impact (Q6: F(2,77) = 16.32, p < .001), interpreting the UV index (Q10: F(2,77) = 8.41, p < .001), and the ABCDE method for skin self-check (Q13: F(2,77) = 44.40, p < .001). Significant time effects were also seen in their ability to assess online content accuracy (Q11: F(2,77) = 3.90, p = .024), understanding of skincare routines (Q16: F(2,77) = 15.87, p < .001), and knowledge of sunburn aftercare (Q15: F(2,77) = 4.26, p = .018). Responses to items related to ethnicity and skin tone (Q12: F(2,77) = 2.90, p = .061) and SPF differences (Q9: F(2,77) = 3.60, p = .032) approached significance.

### Post-hoc analysis by time factor

Bonferroni-adjusted pairwise comparisons revealed meaningful differences between timepoints, highlighting both the immediate impact of the workshop and knowledge retention over time. When comparing pre- and post-workshop responses (Time 1 vs Time 2), students demonstrated significant improvements across key items. These included increased understanding of how UV camera technology visualises skin damage (Q4: Mean Difference = −1.00, p < .001), different sunscreen types and their effectiveness (Q5: −0.96, p < .001), the impact of UV on skin layers (Q6: −1.29, p < .001), the UV index and its application in sun protection (Q10: −0.82, p < .001), strategies to evaluate online health content (Q11: −0.43, p = .046), the ABCDE method for self-skin checks (Q13: −2.21, p < .001), and daily skincare routines for protection and rejuvenation (Q16: −1.18, p < .001). These shifts indicate that the workshop had an immediate and significant effect on students’ knowledge and awareness of sun safety and skin health.

Comparisons between post-workshop and five-month follow-up scores (Time 2 vs Time 3) showed that several learning gains were retained over time. While some items showed slight declines, knowledge levels remained high and statistically significant for sunscreen types (Q5: −0.69, p < .001), UV impact on skin (Q6: −1.00, p < .001), the UV index (Q10: −0.51, p = .032), the ABCDE self-check method (Q13: −1.84, p < .001), and daily protection routines (Q16: −0.76, p < .001). This pattern suggests effective retention of key information well beyond the workshop period. The consistent performance across both phases confirms that the educational intervention not only improved immediate knowledge but also supported longer-term understanding in areas critical to sun safety and skin cancer prevention.

## Discussion

Despite decades of public campaigns and other interventions, skin cancer remains a significant problem in Australia. As most sun exposure occurs before the age of 18, skin cancer prevention and detection messages should be prioritised to educate adolescents on skincare and daily routines that support sun-protective behaviours [26]. This is an important way to approach decreasing the incidence of skin cancer in Australia [27].

This study evaluated the effectiveness of a targeted skin safety school-based workshop, supported by syllabus content, in improving high school students’ understanding of sun protection and skincare. The results show that students’ knowledge significantly improved immediately after the workshop and was retained in key areas after five months. These findings are consistent with prior evidence that school-based interventions can significantly enhance sun safety knowledge and promote healthier behaviours in adolescents [15,28].

This study was conducted in a single independent girls’ secondary school, which represents an important limitation when considering the broader applicability of the findings. While this setting allowed for a tailored, peer-informed, and contextually embedded intervention, the results cannot be assumed to generalise across mixed-gender schools or other educational sectors. Gendered sociocultural influences on tanning norms and appearance likely shaped both baseline attitudes and responses to the intervention. At the same time, focusing on a girls’ school context offers valuable insight into a population known to be at heightened risk due to appearance-related norms, peer influence, and targeted tanning messaging. These findings therefore contribute nuanced evidence to the literature on gender-responsive health education and adolescent sun safety.

The statistically significant time effects across multiple items, including understanding of UV technology, sunscreen types, skin structure, sunburn care, and daily skincare routines, demonstrate that the workshop achieved its primary aim. The largest improvements were observed between the pre- and post-workshop phases, suggesting that direct, structured educational interventions can drive rapid knowledge gains. Items such as the ABCDE self-examination method (Q13) and UV impact on skin layers (Q6) showed particularly large effect sizes and sustained knowledge retention. These outcomes align with prior research advocating for multi-session or curriculum-integrated programs, which have proven more effective than one-off sessions in achieving both short-term and long-term educational outcomes [16,22].

The retention of knowledge over time supports the long-term impact of the intervention. Five months post-workshop, students continued to demonstrate elevated understanding in critical areas such as sun damage detection and skincare practices. This reinforces the value of integrated curriculum initiatives and experiential learning, especially when supported by student-led elements that enhance relevance and engagement [15,22]. Hands-on activities, such as those involving UV dosimeters or peer-led sessions, have been shown to significantly improve learning outcomes by making abstract concepts tangible and empowering students to lead health messaging among peers [22].

The analysis also revealed significant effects of ethnicity. Students from different ethnic backgrounds reported varying levels of understanding, particularly regarding genetic risk (Q1), sunscreen application (Q3), and perceptions of healthy skin tone (Q7). These findings are consistent with research highlighting how sociocultural norms and skin tone ideals influence adolescent tanning attitudes and sun protection behaviours [4,6,10]. Ethnic disparities in sun protection practices have also been linked to lower perceived personal risk and misinformation, particularly among adolescents with richly pigmented skin types [29]. This study’s results therefore suggest that culturally tailored health education is needed to ensure messages resonate equally with all students.

Interestingly, no significant interaction effects were found between time and ethnicity. This suggests that although baseline knowledge differed across groups, all students benefited similarly from the intervention. The workshop appears to offer a broadly inclusive educational strategy. However, to further improve engagement and equity, future iterations should consider adapting content to address specific cultural beliefs and misconceptions about tanning, sunscreen use, and skin cancer risk.

Importantly, the statistically significant differences between pre- and post-intervention scores (rather than just post to delayed) underline the success of the intervention in driving initial change. These gains, coupled with moderate knowledge retention over time, support a two-pronged benefit: short-term impact and sustained awareness. This reinforces calls in the literature for schools to embed evidence-based and recurrent health education on sun safety into the broader curriculum; occasional booster activities serve to strengthen retention and encourage long-term behaviour change [30].

The findings of this study align with prior evaluations of school-based sun safety interventions demonstrating improvements in adolescent knowledge and attitudes following curriculum-integrated education. For example, Geller [14] and subsequent programs reviewed by Calco [15] report consistent short-term gains in sun protection knowledge, although fewer studies examine longer-term retention. In contrast, the present study demonstrated sustained knowledge improvements at five months post-intervention, suggesting that integration within the PDHPE curriculum and the use of interactive workshop approaches may support longer-term learning. Similar to youth-engaged prevention models such as YES-CAN! [22], participants also reported reduced appeal of tanning, an attitudinal shift that has been identified as difficult to achieve during adolescence [9]. However, consistent with previous research, behavioural change remained self-reported, reinforcing the need for cautious interpretation [10,17].

Future research will extend this work through multi-site replication across diverse school contexts, including co-educational schools, government schools, and schools with differing socioeconomic and cultural profiles. Replication will enable comparison of intervention effects across gender compositions and educational settings, strengthen external validity, and support evaluation of scalability. Planned adaptations will retain the core curriculum-aligned and peer-informed structure while allowing contextual flexibility to meet the needs of different school communities.

### Limitations

Several limitations should be considered when interpreting the findings of this study. First, attrition occurred across the three survey timepoints, with 24 of the original 81 participants (approximately 30%) not completing the five-month follow-up survey. While the repeated-measures design ensured matched responses for longitudinal analysis, this level of attrition may limit the representativeness of the final sample and introduce potential non-response bias.

Second, the study relied on self-reported survey data, which are subject to recall bias and social desirability bias. Participants may have over-reported knowledge or sun-protective behaviours following the intervention. Third, while the intervention demonstrated significant and sustained improvements in sun safety knowledge and attitudes, increased knowledge does not necessarily translate into long-term behavioural change. Previous research has shown that adolescents may demonstrate strong awareness of sun safety principles without consistently adopting protective behaviours [2,3]. As such, behaviour change outcomes should be interpreted cautiously.

Finally, as previously advised, the study was conducted in a single independent girls’ school, which may limit generalisability to other school contexts, including co-educational, boys, government, or culturally diverse settings. Future research should replicate the intervention across multiple school types and contexts and incorporate objective or behavioural measures to better assess long-term impact.

## Conclusion

The body of research indicates that with respect to youth, there is a chronic lack of actionability regarding skincare protection and sun safety. Given the importance of skin care protection in guarding against melanoma and that most skin damage occurs in adolescence, it mandates continued efforts for health behaviour change. The results of this research have demonstrated the effectiveness of student-led sun safety messaging within a school curriculum, highlighting both short-term impact and sustained awareness. Further research is needed to validate and refine this intervention across multiple school settings, including mixed-gender and diverse educational contexts, to strengthen the evidence base for broader implementation. In particular, studies could examine whether knowledge gains translate into sustained behaviour change through longitudinal designs incorporating behavioural or observational measures.

## Supporting information

S1 Appendix Pre and post surveys. (DOCX)

S1 File Survey pre, post and 5 month post data set. (PDF)
