# Supplementary material for: A curriculum-integrated sun safety intervention to improve adolescent skin health knowledge in an independent girls’ secondary school
Source: PLoS One. 2026 Jun 26;21(6):e0350659. doi: 10.1371/journal.pone.0350659 (PMC13308783; doi:10.1371/journal.pone.0350659)
Supplement: S1 Appendix — (DOCX) [file pone.0350659.s001.docx]

**Appendix A: Pre and Post Surveys

Can dedicated workshops and associated syllabus implementation be used to improve high school students' knowledge and understanding of skin care and protection?**

**Pre Workshop Survey**

Date ______________ School _____________________

Age: 13 / 14 / 15

Ethnicity: Caucasian, Aboriginal and Torres Strait Islander, African American, Latino or Hispanic, Asian, I would prefer not to say, other unknown

Postcode:

Boarder Y or N

Male, Female, Non-binary, Genderfluid, Agender, Prefer not to say, Other

| **Please rate how much you agree or disagree with the following statements:** | **Strongly Disagree** | **Disagree** | **Neutral** | **Agree** | **Strongly Agree** | **Workshop content** |
| --- | --- | --- | --- | --- | --- | --- |
| 1. I can confidently provide information about the structure of my skin and how sun damage impacts the layers. | **1** | **2** | **3** | **4** | **5** | 1. **Skin Structure and genetics** |
| 1. I am aware of the potential dangers of UV damage on the skin. | **1** | **2** | **3** | **4** | **5** | **1. Skin Structure and genetics**  **1. UV Camera** |
| 1. I understand that skin cancer risk can be passed from family members genetically. | **1** | **2** | **3** | **4** | **5** | **1. Skin structure and genetics** |
| 1. I know how to apply sunscreen to prevent sunburn. | **1** | **2** | **3** | **4** | **5** | **1. UV Camera and**  **2. Sunscreen contents** |
| 1. I can explain how early diagnosis can limit the damaging impact of skin cancer. | **1** | **2** | **3** | **4** | **5** | **1. UV Camera** |
| 1. I know how to apply sunscreen to prevent sunburn. | **1** | **2** | **3** | **4** | **5** | **1. UV Camera and**  **2. Sunscreen contents** |
| 1. I can accurately describe the impact of ultraviolet rays (both UVA and UVB) on my skin. | **1** | **2** | **3** | **4** | **5** | **2. UV Rays** |
| 1. I understand the different types of sunscreen available and how they differ in effectiveness (physical, chemical, broad spectrum: absorb or reflect UVB and UVA rays). | **1** | **2** | **3** | **4** | **5** | **2. Sunscreen contents** |
| 1. I know what Sun Protection Factor (SPF) stands for. | **1** | **2** | **3** | **4** | **5** | **2. Sunscreen contents** |
| 1. I understand what SPF differences mean (e.g. 30 versus 50 SPF). | **1** | **2** | **3** | **4** | **5** | **2. Sunscreen contents** |
| 1. I know what the three main types of skin cancer are (Basal Cell Carcinoma (BCC), Squamous Cell Carcinoma (SCC) and Melanoma). | **1** | **2** | **3** | **4** | **5** | **3. Three types of cancer** |
| 1. I can recognise the three types of skin cancer: Basal Cell Carcinoma (BCC), Squamous Cell Carcinoma (SCC) and Melanoma. | **1** | **2** | **3** | **4** | **5** | **3. Types of skin cancer** |
| 1. I understand the UV index and use it to help protect myself from the sun. | **1** | **2** | **3** | **4** | **5** | **3. Health Literacy** |
| 1. I know how to determine the accuracy of online content regarding sun safety; including on apps and social media. | **1** | **2** | **3** | **4** | **5** | **3. Health literacy** |
| 1. Where do you currently get your information about skincare and skin safety? (choose any or all that apply). | **Social Media (Instagram, TikTok, YouTube)** | **Online sources that I am unsure are accurate** | **Online sources that I know are accurate** | **Medical specialists (doctor, dermatologist, etc.)** | **Talking to peers** | **3. Health literacy** |
| 1. I can confidently explain the methods for performing a self-skin examination using the A (Asymmetry) B (Border) C (Colour) D (Diameter) E (Evolution) method. | **1** | **2** | **3** | **4** | **5** | **4. Medical Consequences** |
| 1. I understand the dangers of tanning at high UV (energy produced by the sun and leads to 95% of all skin cancers). | **1** | **2** | **3** | **4** | **5** | **4. Historical and cultural perspectives** |
| 1. I am aware of ethnicity differences in terms of what a healthy skin colour looks like. | **1** | **2** | **3** | **4** | **5** | **4. Historical and cultural influences** |
| 1. I know that skin types differ in the protection they offer. | **1** | **2** | **3** | **4** | **5** | **4. Historical and cultural influences** |
| 1. I can explain how early diagnosis and the use of technology (skin damage recognition devices such as UV cameras, high-resolution cameras and total body photography) can demonstrate the damaging impact of skin cancer. | **1** | **2** | **3** | **4** | **5** | **5. Emerging technology and early diagnosis** |
| 1. I know what to do after I get a sunburn to help heal quickly. | **1** | **2** | **3** | **4** | **5** | **6. Skin Care station** |
| 1. I know how to implement a daily skincare routine to care for my skin and protect it from sun damage. | **1** | **2** | **3** | **4** | **5** | **6. Skin Care station** |

**Post Workshop Survey**

Date ______________ School _____________________

Age: 13 / 14 / 15

Ethnicity: Caucasian, Aboriginal and Torres Strait Islander, African American, Latino or Hispanic, Asian, I would prefer not to say, other unknown

Postcode:

Boarder Y or N

Male, Female, Non-binary, Genderfluid, Agender, Prefer not to say, Other

| **Please rate how much you agree or disagree with the following statements:** | **Strongly Disagree** | **Disagree** | **Neutral** | **Agree** | **Strongly Agree** | **Workshop content** |
| --- | --- | --- | --- | --- | --- | --- |
| 1. Following the workshop activities, I am able to better understand that skin cancer risk can be passed from family members genetically. | **1** | **2** | **3** | **4** | **5** | **1. Skin structure and genetics** |
| 1. I am more aware of the potential dangers of UV damage on the skin following the workshop activities. | **1** | **2** | **3** | **4** | **5** | **1. Skin Structure and genetics**  **1. UV Camera** |
| 1. I know how to effectively apply sunscreen to prevent sunburn after having completed the workshop activities. | **1** | **2** | **3** | **4** | **5** | **1. UV Camera and**  **2. Sunscreen contents** |
| 1. Following the workshop, I can better understand how technology such as a UV camera is able to show skin damage when it records an image of a person’s skin. | **1** | **2** | **3** | **4** | **5** | **1. UV Camera** |
| 1. Following completion of the workshop, I have improved my understanding of the impact on my skin of UV; both Ultraviolet A (UVA) which is associated with skin ageing, and Ultraviolet B (UVB) which is associated with skin burning. | **1** | **2** | **3** | **4** | **5** | **2. UV Rays** |
| 1. Following completion of the workshop, I can better provide information about the structure of my skin and how sun damage impacts the layers. | **1** | **2** | **3** | **4** | **5** | 1. **Skin Structure and genetics** |
| 1. Since the workshop activities, I can better understand the different types of sunscreen available and how they differ in effectiveness. | **1** | **2** | **3** | **4** | **5** | **2. Sunscreen contents** |
| 1. I am able to better understand what Sun Protection Factor (SPF) stands for now that I have participated in the workshop activities. | **1** | **2** | **3** | **4** | **5** | **2. Sunscreen contents** |
| 1. The workshop activities helped me to better understand what SPF differences mean (e.g. 30 versus 50 SPF). | **1** | **2** | **3** | **4** | **5** | **2. Sunscreen contents** |
| 1. My understanding of the UV index and my ability to use it to help protect myself from the sun have improved after the workshop activities. | **1** | **2** | **3** | **4** | **5** | **3. Health Literacy** |
| 1. I feel more confident since the workshop in using a checklist when deciding the accuracy of online content (including on apps and social media). | **1** | **2** | **3** | **4** | **5** | **3. Health literacy** |
| 1. Following the workshop learning, I will access information about skincare and skin safety from these sources (choose any or all that apply). | **Social Media (Instagram, TikTok, YouTube**) | **Online sources that I am unsure are accurate** | **Online sources that I know are accurate** | **Medical specialists (doctor, dermatologist, etc.)** | **Talking to peers** | **3. Health literacy** |
| 1. I feel more confident since the workshop in using a checklist when deciding the accuracy of online content (including on apps and social media). | **1** | **2** | **3** | **4** | **5** | **3. Health literacy** |
| 1. Having completed the workshop activities, I am more aware of ethnicity differences in terms of what a healthy skin colour looks like. | **1** | **2** | **3** | **4** | **5** | **4. Historical and cultural influences** |
| 1. Having completed the workshop activities, I am more aware of how skin types differ in the protection they offer. | **1** | **2** | **3** | **4** | **5** | **4. Historical and cultural influences** |
| 1. Having learned about the damage of UV to the skin from the workshop, my understanding of the potential dangers of tanning has improved. | **1** | **2** | **3** | **4** | **5** | **4. Historical and cultural perspectives** |
| 1. Since my workshop participation, I can better explain the methods for performing an ABCDE (Asymmetry) B (Border) C (Colour) D (Diameter) E (Evolution) method of self-check. | **1** | **2** | **3** | **4** | **5** | **4. Medical Consequences** |
| 1. Following the workshop, I can explain more comprehensively how early diagnosis can limit the damaging impact of skin cancer. | **1** | **2** | **3** | **4** | **5** | **5. Emerging technology and early diagnosis** |
| 1. I have a better understanding of what to do after I get a sunburn to help heal quickly following the workshop activities. | **1** | **2** | **3** | **4** | **5** | **6. Skin Care station** |
| 1. Having completed the workshop activities, I now understand the steps of a daily skincare routine to care for my skin. | **1** | **2** | **3** | **4** | **5** | **6. Skin Care station** |
| 1. The workshop activities have increased my understanding of good daily skin protection routines I can use to protect and rejuvenate my skin. | **1** | **2** | **3** | **4** | **5** | **6. Skin Care station** |

## **Healthy, Safe and Active Lifestyles**

### Outcomes

**A student:**

› examines and demonstrates the role help-seeking strategies and behaviours play in supporting themselves and others PD4-2

› recognises how contextual factors influence attitudes and behaviours and proposes strategies to enhance health, safety, wellbeing and participation in physical activity PD4-6

› investigates health practices, behaviours and resources to promote health, safety, wellbeing and physically active communities PD4-7

› plans for and participates in activities that encourage health and a lifetime of physical activity
PD4-8

› demonstrates self-management skills to effectively manage complex situations PD4-9

› applies and refines interpersonal skills to assist themselves and others to interact respectfully and promote inclusion in a variety of groups or contexts PD4-10

**Related Life Skills outcomes:** PDLS-2, PDLS-3, PDLS-7, PDLS-8, PDLS-9, PDLS-10, PDLS-11

Specific Syllabus links

1. **What positive actions contribute to the health, safety, well-being and participation in physical activity levels of the wider community?**

• recognise potentially unsafe environments and describe strategies to promote their own and others’ health, safety and wellbeing in a variety of
 real-life situations

− propose strategies individuals and others can use to make safe and informed decisions S I

− recognise how different cultures, including Aboriginal and Torres Strait Islander and Asian cultures, value the contribution of the mind
 body–spirit connection to health and wellbeing

• develop health literacy skills and promote health information that is aimed at assisting young people to address health issues

− assess health products, information and advertising to expose myths and fallacies, e.g. understanding food labels, contraceptive
 products S

1. **What positive actions contribute to the health, safety, wellbeing and participation in physical activity levels of the wider community?**

• develop health literacy skills and promote health information that is aimed at assisting young people to address health issues

− assess health products, information and advertising to expose myths and fallacies, e.g. understanding food labels, contraceptive
 products S

− evaluate the credibility of media messages conveyed by different sources in terms of bias, reliability and validity S

− promote and apply credible health messages and information to positively influence the decisions of their peers and the wider
 community S I

1. **What positive actions contribute to the health, safety, wellbeing and participation in physical activity levels of the wider community?**

• plan and use health practices, behaviours and resources to enhance the health, safety, wellbeing and physical activity participation of their communities (ACPPS077)

− design and implement health promotion activities, using ICT tools as appropriate, targeting preventive health practices relevant to
 young people S I

1. **How can I effectively manage my own and support others’ health, safety, wellbeing and participation in physical activity?**

• propose and develop protective strategies to effectively manage their own personal health, safety and wellbeing (ACPPS073)

− examine and develop strategies for safe practices in outdoor environments
