# Supplementary material for: A curriculum-integrated sun safety intervention to improve adolescent skin health knowledge in an independent girls’ secondary school
Source: PLoS One. 2026 Jun 26;21(6):e0350659. doi: 10.1371/journal.pone.0350659 (PMC13308783; doi:10.1371/journal.pone.0350659)
Supplement: S1 File — (PDF) [file pone.0350659.s002.pdf]

Supplementary Data File: Survey Pre, Post and 5 Month Post Data Set

ID	Ethnicity	Factor	PostQ1	PostQ2	PostQ3	PostQ4	PostQ5	PostQ6	PostQ7	PostQ8	PostQ9	PostQ10	PostQ11	PostQ12	PostQ13	PostQ14	PostQ15	PostQ16
1	0	1	3	3	2	3	1	1	3	3	3	4	3	3	0	3	3	1
2	1	1	3	3	3	2	2	0			2	2	2	2	2	0	2	2
3	2	1	3	3	2	3	2	0	3	3	3	0	4	3	0	3	2	1
4	2	1	3	4	3	1	1	3	4	3	3	2	3	3	1	1	3	3
5	1	1	2	4	4	3	4	1	3	4	4	4	4	4	0	4	4	3
6	0	1	4		4	3	3	4	4	4	3	3	4		2	4	4	3
7	0	1	4	4	4	3	3	2	2	4	4	4	4	2	2	4	4	2
8	0	1	4	4	4	3	4	1	4	4	4	4	4	4	1	4	4	3
9	0	1	4	4	3	2	2	3	4	4	3	2	3	3	1	2	2	3
10	1	1	4	3	4	3	2	3	4	4	3	2	3	3	1	3	2	2
11	0	1	3	4	4	4	3	3	4	4	2	1	2	4	0	3	4	1
12	2	1	3	3	3	2	2	3	3	3	3	2	3	2	0	3	3	3
13	0	1	2	3	3	2	1	1	3	3	3	2	3	4	0	2	3	1
14	1	1	3	4	3	2	4	3	3	2	3	3	3	3	2	4	4	3
15	0	1	4	4	4	1	2	4	4	4	4	2	4	4	0	2	4	3
16	0	1	4	4	3	3	3	2	3	3	3	2	3	2	2	4	3	2
17	2	1	4	4	4	3	2	2	4	4	4	3	4	3	4	3	4	4
18	1	1	2	2	2	2	2	2	2	2	2	2	2	2	2	2	2	2
19	2	1	4	4	4	3	3	4	4	4	4	4	3	3	2	3	3	4
20	2	1	4	4	4	4	2	3	4	4	3	3	4	4	2	4	3	3
21	0	1	4	4	4	3	3	4	4	4	4	4	4	4	3	4	3	3
22	1	1	3	3	3	2	3	2	3	3	3	3	3	3	3	2	3	3
23	1	1	4	4	3	3	3	3	3	3	3	3	4	3	3	4	3	3
24	0	1	4	4	4	3	3	4	4	4	4	4	3	4	2	4	3	3
25	0	1	3	4	3	4	3	1	3	2	4	4	2	4	1	4	4	1
26	0	1	3	3	3	1	3	3	3	3	3	3	3	3	0	3	3	2
27	2	1	3	3	3	2	3	3	3	3	3	3	3	3	1	3	3	3
28	2	1	4	3		2	3	4	4	4	3	2	3	2	1	2	4	3
29	1	1	3	4	4	2	3	2	3	4	3	4	3	3	3	3	4	3
30	0	1	4	4	4	4	4	3	3	3	3	3	4	4	2	4	4	3
31	0	1	4	4	4	2	4	2	4	4	3	3	3	4	3	3	3	3
1	0	2	4	4	4	4	4	4	4	4	4	4	4	4	4	4	4	4
2	1	2	3	3	3	3	3	3	3	3	3	3	3	3	3	3	3	3
3	2	2	4	4	4	4	4	4	4	4	4	4	4	4	4	4	4	4
4	2	2	4	4	4	4	4	4	4	4	4	4	4	4	4	4		4
5	1	2	4	4	4	4	4	4	4	4	4	4	4	4	4	0	4	4
6	0	2	4	4	4	4	4	4	4	4	4	4	4	4	4	4	4	4
7	0	2	4	4	4	4	4	3	3	4	4	4	4	4	4	4	4	4
8	0	2	4	4	4	4	4	4	4	4	4	4	4	4	4	2	4	4
9	0	2	4	4	4	4	4	3	4		3	4	3	3	3	3	2	2
10	1	2	4	4	4	4	3	4	4	4	4	3	3	3	3	2	3	3
11	0	2	4	4	4	4	4	4	4	4	4	4	4	4	4	2	4	4
12	2	2	4	4	4	4	3	4	4	4	4	4	4	4	4	2	4	4
13	0	2	3	3	3	3	3	3	3	3	3	3	3	3	3	3	3	3
14	1	2	4	4	4	4	3	4	3	4	4	4	4	4	4	4	4	4
15	0	2	4	4	4	2	4	4	4	4	4	4	3	4	4	2	4	4
16	0	2	4	4	4	4	4	4	4	4	4	4	4	4	4	4	4	4
17	2	2	4	4	4	4	4	4	4	4	4	4	4	4	4	4	4	4
18	1	2	2	2	2	2	2	2	2	2	2	2	2	2	2	2	2	2
19	2	2	3	3	3	3	3	3	3	3	2	3	3	3	3	3	4	4
20	2	2	4	4	4	4	4	4	4	4	4	4	4	4	4	4	4	4
21	0	2	4	4	4	4	4	4	4	4	4	4	4	4	4	4	4	4
22	1	2	3	3	2	3	4	4	3	4	4	3	4	3	4	3	3	4
23	1	2	3	3	3	3	3	3	3	3	3	3	3	3	3	3	3	3
24	0	2	4	4	4	4	4	4	4	4	4	4	4	3	3	4	4	4
25	0	2	4	4	4	4	4	4	4	4	4	4	4	4	4	4	4	4
26	0	2	3	3	3	3	3	3	3	3	3	3	3	3	3	3	3	3
27	2	2	4	4	4	4	4	4	4	4	4	4	4	4	4	4	4	4
28	2	2	4	4	4	4	4	4	4	4	4	4	4	4	4	4	4	4
29	1	2	4	4	4	3	4	4	3	4	3	4	4	3	4	3	4	3
30	0	2	4	4	4	4	4	4	4	4	4	4	4	4	4	4	4	4
31	0	2	4	4	4	4	4	4	4		4	4	4	4	4	4	4	4
1	0	3	3	3	3	3	3	1	3	3	3	3	2	2	2	1	1	2
2	1	3	3	3	3	3	3	3	3	3	3	3	2	2	2	2	2	2
3	2	3	4	4	3	3	3	3	4	4	4	3	4	4	4	4	4	4
4	2	3	3	3	3	3	3	3	3	3	3	3	3	3	3	3	3	3
5	1	3	4	4	4	4	4	4	4	4	4	4	4	4	4	2	4	4
6	0	3	4	4	4	4	4	4	4	4	4	4	4	4	4	4	3	3
7	0	3	4	4	4	3	4	4	4	3	4	4	4	3	4	4	4	4
8	0	3	4	4	4	4	3	4	4	4	4	3	3	4	3	2	4	4
9	0	3	4	4	3	4	4	3	4	4	4	4	4	3	3	2	3	4
10	1	3	3	3	3	3	3	3	3	4	3	3	3	3	3	2	3	3
11	0	3	4	4	4	4	2	4	4	4	4	3	3	4	3	2	4	4
12	2	3	4	4	4	3	3	3	4	4	4	3	3	3	4	4	4	3
13	0	3	4	4	4	4	4	3	4	4	3	3	4	3	4	4	3	2
14	1	3	3	4	4	3	4	4	3	3	3	4	2	2	3	2	4	3
15	0	3	4	4	4	3	3	4	4	4	3	4	2	4	4	4	4	4
16	0	3	3	4	3	3	3	3	3	3	3	3	3	3	3	3	3	3
17	2	3	4	4	4	4	4	4	4	4	4	4	4	4	4	4	4	4
18	1	3	2	2	2	2	2	2	2	2	2	2	2	2	2	2	2	2
19	2	3	3	3	3	3	3	3	3	3	3	3	3	3	3	3	3	3
20	2	3	4	4	4	4	4	4	4	4	4	4	4	4	4	4	4	4
21	0	3	4	4	4	4	4	4	4	4	4	4	4	4	4	4	4	4
22	1	3	4	4	4	3	4	3	2	3	4	3	4	3		4	4	3
23	1	3	3	3	4	3	3	4	3	3	4	3	3	4	3	3	3	3
24	0	3	3	4	4	3	4	4	4	4	4	4	4	4	4	4	3	3
25	0	3	4	4	4	4	3	3	3	3	3	3	3	3	3	3	3	3
26	0	3	4	3	3	4	3	4	4	4	3	3	4	3	3	3	4	3
27	2	3	4	4	4	4	4	4	4	4	4	3	3	4	4	2	4	4
28	2	3	3	4	4	4	3	4	4	4	4	4	3	2	2	2	4	4
29	1	3	4	4	3	4	3	4	4	4	4	3	4	4	4	4	4	3
30	0	3	4	4	4	4	4	4	4	4	3	4	3	4	3	4	3	3
31	0	3	4	4	4	3	4	2	3	4	3	3	4	3	3	3	4	4

	PostQ1	PostQ2	PostQ3	PostQ4	PostQ5	PostQ6	PostQ7	PostQ8	PostQ9	PostQ10	PostQ11	PostQ12	PostQ13	PostQ14	PostQ15	PostQ16
Avg	3.419355	3.6	3.4	2.580645	2.677419	2.451613	3.4	3.433333	3.193548	2.806452	3.225806	3.166667	1.483871	3.032258	3.225806	2.548387
Stdev	0.672022	0.563242	0.674665	0.847514	0.871286	1.178663	0.621455	0.678911	0.601074	1.013882	0.668814	0.74664	1.15097	1.016001	0.716923	0.850047
Avg	3.741935	3.741935	3.709677	3.645161	3.677419	3.709677	3.645161	3.758621	3.677419	3.709677	3.677419	3.612903	3.677419	3.225806	3.666667	3.677419
Stdev	0.514311	0.514311	0.588419	0.608188	0.540808	0.528744	0.550659	0.510964	0.599283	0.528744	0.540808	0.558416	0.540808	0.990275	0.606478	0.599283
Avg	3.612903	3.709677	3.612903	3.451613	3.387097	3.419355	3.548387	3.612903	3.516129	3.354839	3.290323	3.290323	3.3	3.032258	3.419355	3.290323
Stdev	0.558416	0.528744	0.558416	0.567962	0.615219	0.764818	0.623897	0.558416	0.569852	0.550659	0.739078	0.739078	0.702213	0.948116	0.764818	0.69251

Factor 1 Pre-workshop
Factor 2 Post-workshop
Factor 3 Five-month post-workshop
